# CGAT: a model for immersive personalized training in computational genomics

**DOI:** 10.1093/bfgp/elv021

**Published:** 2015-05-16

**Authors:** David Sims, Chris P. Ponting, Andreas Heger

**Keywords:** training, genomics, bioinformatics, next-generation sequencing, integrative biology

## Abstract

How should the next generation of genomics scientists be trained while simultaneously pursuing high quality and diverse research? CGAT, the Computational Genomics Analysis and Training programme, was set up in 2010 by the UK Medical Research Council to complement its investment in next-generation sequencing capacity. CGAT was conceived around the twin goals of training future leaders in genome biology and medicine, and providing much needed capacity to UK science for analysing genome scale data sets. Here we outline the training programme employed by CGAT and describe how it dovetails with collaborative research projects to launch scientists on the road towards independent research careers in genomics.

## Introduction

The acute skills shortage in computational biology is widely acknowledged [1] and has been greatly magnified by the advent of more affordable genomic technologies, in particular next-generation sequencing. There have been numerous efforts to address this shortage by providing short-term training courses for early and mid career scientists [2, 3], by developing Masters courses in Bioinformatics, and by initiatives from funding bodies (UK MRC, BBSRC) to provide fellowships to bring individuals from numerical disciplines (mathematics, physics, engineering) into the life sciences.

While short courses can provide an excellent introduction to a subject area, they are not able to offer real-world experience [4]. Time constraints and the steepness of the learning curve mean that frequently only a single approach to a particular analysis problem can be covered. Attendees are usually not able to work with their own data sets and methods learnt on a course may not be the most appropriate for their particular question. This approach can only enable biologists to use existing bioinformatics tools and resources rather than equip them to answer any question, whether appropriate tools exist or not. Furthermore, relevant courses are often not available or are heavily over-subscribed.

Masters programmes offer more in-depth training, allowing students time to build up a core set of bioinformatics skills [5], and offering a chance for project-based learning. However, these courses are targeted at early career scientists, often without first-hand experience of biological research. It can, on one hand, be disadvantageous in the short term for career scientists to take a full year out of their research career to focus entirely on taught-course training. On the other hand, the acquisition of valued skills can transform a career and allow individuals to stand out from their peers.

Over the past 10 years, there has been a welcome and much needed influx of physicists and mathematicians into biology, and their associated skills have been influential in the development of novel computational tools (for example, read aligners, assemblers) and statistical approaches (differential gene expression from RNA-seq data) to address major computational problems in genomics. However, maximizing the impact of genomic data sets on important biomedical problems needs to go beyond method development and requires framing an innovative hypothesis, analysing data synthetically using multiple tools, and interpreting results biomedically in an iterative fashion. It is at this juncture where the shortage of researchers with equally deep know-how in data science and in biology is most acute. Such individuals will rarely graduate from a first degree if only because the modern biological syllabus is equally as broad as one in statistics or computational science, and an undergraduate’s learning capacity will always be finite.

## The CGAT training approach

The Computational Genomics Analysis and Training programme (CGAT, http://cgat.org) is founded on the premise that many genomic projects stall once standard processing of genomic data has completed. This bottleneck is a direct consequence of a shortage of individuals who can draw both on their biological knowledge to ask the pertinent questions, and on their computational skills and statistical knowledge to perform the relevant analyses, thereby ensuring that the results of their analyses are interpreted appropriately.

The CGAT programme recruits numerate biologists or medics with a strong motivation to acquire deep computational and statistical skills. Fellows are recruited in pairs every 6 months. CGAT fellowships are advertised widely and candidates are asked to submit a curriculum vitae and supporting statement as part of the selection process. Shortlisted candidates are invited to attend a formal interview, where they are asked to give a presentation on how a CGAT training fellowship would enhance their scientific career. During this process, we evaluate career trajectory, numeracy, scientific thinking and drive. We seek evidence that candidates have come into contact with large-scale data sets and that they have shown the motivation to begin teaching themselves basic skills. This motivation has often been born of the recruit’s frustration when unable to proceed beyond the data analysis and interpretation bottleneck. The programme provides these individuals with the skills in computation and statistics necessary for them to design and interpret genomic experiments relevant to their chosen field of interest. CGAT trains post-doctoral scientists so as to ensure that candidates have already attained sufficiently deep biological knowledge and experience in laboratory research to permit them to design and execute experiments.

## The CGAT training model

The CGAT model is one of trainee-led, deep, immersive training. CGAT training fellowships last for a maximum of 3 years and are structured into three phases: an initial assessment and basic training period, followed by project-based training and finally a collaborative research project led and executed by the trainee.

### Initial assessment and basic training

**Figure 1:**
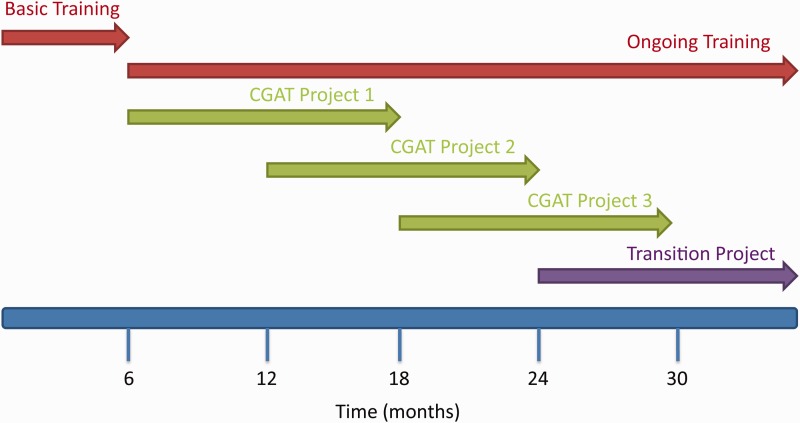
A Gantt chart indicating the structure of a typical CGAT fellowship. Fellows start with up to 6 months of basic training before starting their first project. Fellows work on several overlapping projects during the course of their training and have an opportunity to design and implement their own project (transition project) in the final year of their fellowship. (A colour version of this figure is available online at: http://bfg.oxfordjournals.org)

On arrival, CGAT fellows complete a self-assessment form containing more than 250 questions covering the domains of programming, computer science, statistics, bioinformatics, system administration and genome biology. Based on this initial self-assessment, the fellows and CGAT trainers together develop a personalized training plan so that fellows eventually acquire a core set of computational skills, such as computing within a unix high-performance computing environment, programming/scripting, databases and version control [5]. The exact nature and length of this initial phase varies depending on the experience of the individual, but typically is 3–6 months (Figure 1).

Much early focus of training is on programming. We use Python [6] because it is easy to learn, widely used and open-source [7]. In the initial training phase, the fellows make use of a range of self-study materials including textbooks, online courses [8, 9] (e.g. EdX [10], Coursera [11]) and exercises (e.g. Rosalind [12]) as well as in-house materials. As fellows progress, they are challenged by more complex scripting exercises with genomics relevance in order that they might develop algorithmic thinking and be introduced to current problems in genomics. During this process fellows are guided towards adoption of a common coding style and software engineering best-practices [13–15]. Fellows receive regular code reviews from trainers to help develop their skills. Once fellows are sufficiently confident writing their own scripts to answer biological questions, they learn to create pipelines to analyse large-scale data sets and to generate automated reports for reproducible research [16].

CGAT shares a central code repository [17] that contains hundreds of scripts designed for the analysis of genomic data sets and is publically available on GitHub (https://github.com/CGATOxford). All scripts, modules and pipelines follow a common style guide and incorporate unit tests and regression tests to ensure accurate and consistent functionality. CGAT fellows participate in a biannual release process where effort is focussed on ensuring that released scripts are fully documented, thoroughly tested and share a standard interface. This provides a valuable learning experience for new fellows, enabling them to become familiar with the diverse array of tools already implemented, while established fellows acquire the skills to manage and sustainably develop a large codebase under collaborative development. Indeed a recent review from the Software Sustainability Institute [18] praised CGAT code development practices highly.

### Training by doing—CGAT projects

Once fellows attain proficiency in unix and Python, they progress to the second phase of the fellowship: project-based learning (Figure 1). This apprenticeship phase of the CGAT programme challenges fellows to analyse and interpret real genomic data sets. This approach allows fellows to address important biological questions and to develop robust approaches for data integration, interrogation, interpretation and mining [19]. CGAT collaborates with scientists across the UK to analyse and interpret genome-scale data sets to answer important questions in genomic medicine. We select projects where fellows can make a substantial contribution to the biological interpretation of the question being investigated, and where the data analysis provides a novel and challenging piece of work for the fellow [20–28].

As a consequence, CGAT fellows provide a substantial contribution to these collaborative projects, which is appropriately acknowledged with (joint) first author publications. Indeed, this authorship model is stipulated by CGAT when the collaboration is first formalized. Only when a collaboration produces an unanticipated research direction that the fellow cedes to another researcher, will this authorship model be modified. This model simultaneously provides: (i) deep training of fellows using real data in cutting-edge biological research, (ii) pre-existing computational genomics expertise from the CGAT scientific environment to be brought to bear on the biological question and (iii) the fellow with the (joint) first author publication that is often required to progress their scientific career. At the same time, it allows collaborating groups to undertake projects for which they lack the computational skills and genomic expertise. Projects typically last for 6–12 months, but often contain phases of inactivity when data are being generated or hypotheses are being investigated experimentally by collaborators. As a result, fellows often work on two or more projects simultaneously. This multi-tasking provides useful experience in time management and project management skills that will benefit fellows throughout their career.

Fellows take the lead on computational analysis and interpretation and manage all communication with the collaborators. This provides fellows with essential experience in managing scientific collaborations through both successful and trying periods. CGAT supports and mentors fellows in the background and encourages good working practice in the organization and documentation of computational experiments [29] so that a person unfamiliar with a project can understand what was done and why.

### Ongoing training and assessment

**Figure 2:**
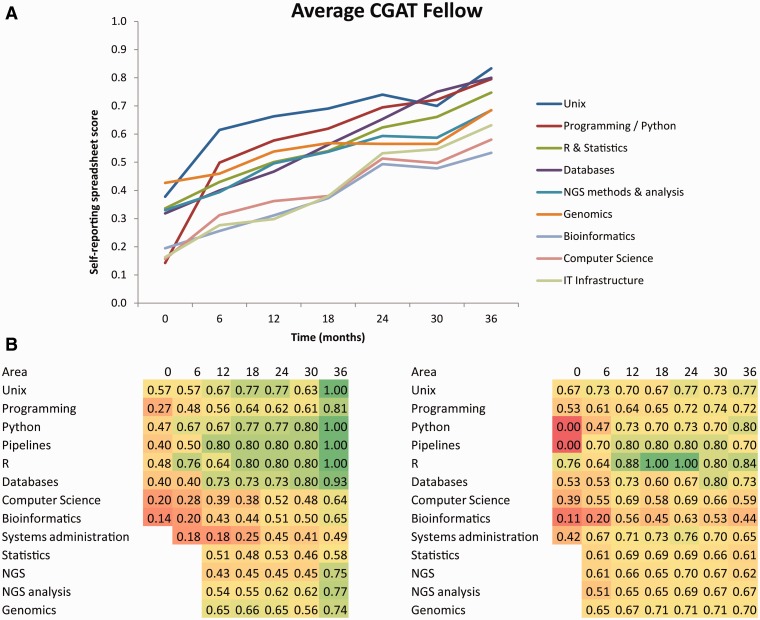
The effect of CGAT training based on self-reporting from CGAT fellows using a 250-point questionnaire. Fellows are asked to indicate their knowledge, experience and confidence in each area on a 5-point scale. Here the scores have been normalized to lie in a range between 0 and 1. (**A**) Average scores for different training areas for nine CGAT fellows with more than 2 years training. (**B**) Heatmaps of changes in scores over time (0–36 months) for two individual CGAT fellows who joined with different levels of experience, from self-taught (left) to novice (right) and who selected their own training emphasis over their time in CGAT. Blank squares indicate missing data due to changes in the questionnaire. (A colour version of this figure is available online at: http://bfg.oxfordjournals.org)

Formal training continues through the 3 years of the fellowships (Figures 1 and 2a). Fellows manage an individual training budget from which they fund external training opportunities such as courses, workshops and conferences. Fellows have previously used such funds to attend statistics courses at the Open University, Software Carpentry Bootcamps, EBI / Wellcome Trust advanced training courses and the University of Washington Summer Institute in Statistical Genetics. Fellows are also able to take advantage of training courses within the University of Oxford, and those provided by the Medical Research Council in areas such as project management and leadership. The choice of courses and training materials is driven by the existing skills and interests of each fellow, in consultation with trainers, such that each fellow’s time in CGAT is different (Figure 2b).

Within CGAT we hold a weekly journal club, which covers both new developments in genomics, to ensure that fellows keep up-to-date with the broader field, and landmark papers in the field, so that everyone appreciates the history of the field. Although most training is self-led, we also hold regular teaching sessions, led by CGAT trainers and associate trainers, addressing both specific and general topics; these have included molecular evolution and population genetics as well as parallelization, object-oriented programming and information theory.

Throughout their time in CGAT, fellows attend scheduled monthly meetings with senior staff to plan and review their training. To monitor their training progress, the fellows update their skills assessment spreadsheet every 6 months. A strong emphasis is placed on the development of general, transferable skills of managing oneself, a project and a group of collaborators. In addition to attending workshops on scientific leadership [30, 31], project management and scientific communication [32] at the MRC and Oxford University, fellows have regular scheduled meetings with the director (C.P.P.) to discuss personal and career development.

### Towards independence—the CGAT transition project

As CGAT fellows’ training may take them away from their main area of biological interest, we consider it important for them to be assisted in their transition towards independent research in their chosen field towards the end of their fellowship. In their final year, CGAT fellows are presented with the opportunity to design their own research project (Figure 1). They are required to contact potential collaborators and with them design a genomics experiment that addresses a key question in their field, or provides pilot data for a future fellowship or grant application. Fellows are asked to submit a grant proposal to CGAT, detailing the background, experimental design and sequencing costs for the project. If their project passes internal review, they are awarded limited funds for next-generation sequencing on that project. This process is intended to provide the fellow with valuable experience in establishing fruitful collaborations, grant writing, project planning and budgeting as well as exposing them to a grant reviewing system.

### Training as a cohort

One of the attractive features of CGAT is the unique scientific environment. CGAT fellows are part of a cohort of eight MRC career development fellows, who are recruited in pairs every 6 months and share one office. This means that there are always others around who have experienced the same learning curve and can offer help and advice. CGAT sits alongside two well-established computational biology groups led by Prof Chris Ponting and Dr Caleb Webber, with whom weekly lab meetings are shared. This creates a super-group of over 40 computational biologists working on a diverse array of subjects. CGAT also has close links with the MRC Functional Genomics Unit and the Department of Physiology, Anatomy and Genetics in which it is hosted. CGAT has strived to create broader links across the University and beyond. We work closely with several MRC sequencing hubs (the Centre for Genomic Research in the University of Liverpool, Edinburgh Genomics and Oxford Genomics). We co-host a monthly special interest group on next-generation sequencing that attracts scientists from across the University of Oxford, and provides CGAT fellows with an opportunity to present their work to a wider audience. CGAT fellows host an annual seminar series for which they have the opportunity to invite speakers from across the UK to speak about their work. Indeed, in 2014, CGAT hosted the Genome Science: biology, technology and bioinformatics conference (www.genomescience.org.uk), and fellows contributed towards the organization and running of the event.

### Training the trainers

By training fellows who will go on to leadership positions in computational genomics, we aim to maximize our impact by producing the next generation of trainers. As fellows move on and become independent, they can impart the skills and practices learnt at CGAT to their post-docs and PhD students. To encourage this, we offer fellows the opportunity to gain both training and experience in teaching [2]. In the past few years, fellows have taught courses in R programming within the University of Oxford and CGAT has organized and delivered an EBI advanced course in the interpretation of next-generation sequencing experiments.

We keep close contact with CGAT alumni, inviting them back for our annual retreat to talk to current fellows about making the transition to independence. We also keep them actively involved in developing the CGAT code repository and encouraging others to contribute to make it a growing, up-to-date resource for reproducible and open-access genomics analysis.

## Conclusions and future perspectives

CGAT is a model of post-doctoral training for researchers extending their expertise into new scientific fields. We aim to first establish sound computational skills, then broaden scientific and genomics experience through a range of different collaborative projects, before allowing trainees to focus on their particular area of interest and lead a project from design to publication. All the while we provide mentorship, support good working practice and continue developing fellows’ technical, analytical, management and leadership skills. By establishing critical mass in a given field and immersing trainees in a dynamic scientific environment with a cohort of fellows undergoing a similar journey, we aim to provide deep and broad training in computational genomics that will give fellows the academic credentials to go on to become leaders in the field. This is a field in which truly qualified individuals are in short supply, and we maximize our impact if CGAT fellows go on to train others in what they have learnt. Consequently, our success will be measured not only by the number of successful collaborations we establish, and the high-impact peer-reviewed publications that result from them, but also from the career paths of our alumni. Since the start of CGAT, three fellows have completed the programme. Each contributed to at least four published peer-reviewed articles, including reviews and methods papers. They are also all still working on at least one CGAT project (the transition project), which we expect to lead to one or more publications. Thus far demand for CGAT graduates has been extremely high and all of our graduating fellows with previous post-doctoral experience have left to take on leadership positions. Indeed, another fellow left to take up a lectureship before completing 3 years in the programme. The remaining graduate, who joined CGAT directly following the completion of his PhD, is currently in a transitional post-doctoral position and applying for fellowships to become an independent researcher.

CGAT was conceived to complement existing short-term training courses and master degrees in bioinformatics as a means of training biologists in computational methods. The model is intended to fill a particular niche not catered for by existing post-doctoral training programmes: offering talented numerate biologists the opportunity to acquire new computational and statistical skills while immersed in a thriving research environment. There is certainly no shortage of qualified individuals interested in participating in the training we offer, as evidenced by the 80–100 applications we receive for each round of recruitment. Naturally, our deep training model cannot provide the throughput offered by shorter courses. However, we believe that 3 years at a postdoctoral level are the minimum required to produce genomicists, who are equally at home at both the bench and the computer, and are able to take an idea from conception, through experimental design and analysis to publication. In the long term, one should consider the throughput of such training models to include those trained by alumni who have gone on to be leaders in the field.

We are also striving to expand the number of individuals who benefit from CGAT training without endangering the success of the current model. To this end, we are piloting a short-term training initiative where newly recruited fellows undergo basic training remotely before joining CGAT for 6 months to work on the analysis and interpretation of their own project. After this period of face-to-face project-based training is completed, fellows return to their host institution but are still mentored by CGAT trainers. We believe that this new model can leverage the critical mass already established in CGAT without detracting from the existing programme. We are also investigating the feasibility of placing our training materials online [33, 34] to create a computational genomics curriculum that is open to all. While much of the CGAT training is embedded in an active research environment that cannot be reproduced online, there are many commonalities in the training paths followed by CGAT fellows that could be captured to create a flexible and modular curriculum that others could follow. Such a curriculum would acknowledge the many excellent Massive Open Online Courses (MOOCs) and exercises already available on the internet, and would serve as a guide through the myriad training possibilities available online.

CGAT’s focus is on computational genomics, but its apprenticeship model is general and can be adopted in other inter-disciplinary fields in which highly trained experts in one discipline need to acquire deep skills in an unrelated field.

CGAT undergoes annual external review from its scientific advisory board to ensure that we continue to implement best practice and meet the demands of 21st-century biomedical research community. As we move forward we are increasingly focusing on the fields of big data and genome medicine. This is because, for example, of the unprecedented demand resulting from the UK’s 100 000 genomes project for computationally trained clinical scientists and healthcare professionals who can accurately interpret genomic data sets.

**Key points**

- There is currently a critical skills gap in the analysis and interpretation of genomic data sets.
- CGAT is an initiative designed to train numerate biologists in computational genomics.
- CGAT combines skills-based learning with real-world genomics project research to provide deep, long-term training.
